# The attitudes of 1066 patients with cancer towards participation in randomised clinical trials

**DOI:** 10.1038/sj.bjc.6606004

**Published:** 2010-11-30

**Authors:** V Jenkins, D Farewell, L Batt, T Maughan, L Branston, C Langridge, L Parlour, V Farewell, L Fallowfield

**Affiliations:** 1 Cancer Research Psychosocial Oncology Group, Brighton & Sussex Medical School, University of Sussex, Sussex, UK; 2 Cardiff University School of Medicine, Cardiff, UK; 3 NISCHR CRC/Wales Cancer Research Network Cardiff, Cardiff, UK; 4 MRC Biostatistics Unit, Institute of Public Health, University Forvie Site, Cambridge, UK

**Keywords:** patients’ attitudes, RCTs

## Abstract

**Background:**

Barriers to randomised clinical trial (RCT) recruitment include failure to identify eligible patients, reluctance of staff to approach them and attitudes of some health-care professionals and patients. As part of a larger UK prospective study examining the communication and involvement in RCTs of 22 multidisciplinary teams in Wales, we also assessed the attitudes of patients they treat towards trials.

**Methods:**

Out of 1146 patients attending outpatient departments who were approached, 1146 (93%) completed the seven-item Attitudes to Randomised Trials Questionnaire (ARTQ), probing their general attitudes towards medical research and likely participation in a hypothetical two-arm RCT.

**Results:**

Randomisation initially deterred many patients from endorsing a willingness to participate. However, if information about the trial logic, voluntary nature and rights to withdraw were provided, together with further treatment details, 83% (886 out of 1066) would potentially participate. Other variables associated with a positive inclination towards participation included previous trial experience (*P*<0.01), male gender (*P*<0.01) and younger age, with patients ⩾70 years less likely to consider trial entry (*P*<0.01).

**Conclusion:**

The majority of patients were receptive to RCT participation. Many of those initially disinclined because of randomisation would consider joining if given further details that form part of standard GCP consent guidelines. These data show the importance and need for clear communication and information to encourage RCT participation. Evidence-based training courses are available to assist with this.

Worldwide, the number of potentially eligible patients recruited to clinical trials is low, impeding accrual of the research evidence to inform and improve clinical practice. The UK clinical research networks aim to augment quality, relevance and focus of research in the National Health Service (NHS) and, since its inception, recruitment to cancer clinical trials has significantly improved.

Some trials recruit better than others, and the features associated with this have been examined. Factors linked to good and poor recruitments were explored in a cohort of 114 randomised clinical trials (RCTs) that had been recruiting participants in the United Kingdom between 1994 and 2002 (McDonald *et al*, 2006). Almost a third of trials (31%) managed to recruit their original target sample size, whereas a similar proportion (31%) failed to do so. Even when investigators were given a time extension, the proportion achieving targets improved only slightly. Many trials (41%) were delayed in starting and 63% had problems at an early stage in the recruitment process. The barriers found were similar to those identified in previous studies: funding issues, changes in the hospital administrative systems, fewer eligible patients than anticipated, problems inherent to some trial types/methodology, (e.g., placebo control), no treatment arms and the difficulties of describing randomisation (McDonald *et al*, 2006; White *et al*, 2008a, 2008b). Additionally, the attitudes of both health professionals and patients towards RCTs have a profound influence upon successful trial recruitment and participation (Fallowfield *et al*, 1997, 1998; Ulrich *et al*, 2010).

The extra time involved in discussing a trial in busy NHS clinics undoubtedly hampers recruitment, and some health-care professionals also introduce idiosyncratic eligibility criteria when deciding which patients are even offered trials (Jenkins *et al*, 1999). Consequently, innovative workshops and educational materials have been developed that have been shown to facilitate health professionals’ discussions of trials (Jenkins *et al*, 2005).

Researchers from various disciplines have examined patients’ expectations and understanding about different aspects of the consent procedure in an effort to identify ways to improve the process (Burnet *et al*, 2004; Wright *et al*, 2004; Markman *et al*, 2006; Bergenmar *et al*, 2008). Interventions have tried to increase patients’ knowledge about clinical trials, including the use of audiovisual aids (Hutchison *et al*, 2007) or mass marketing campaigns (Umutyan *et al*, 2008). Despite all these efforts, a better understanding of patients’ attitudes might encourage health-care professionals to approach more of their eligible patients and also help refine the way they discuss the trial-relevant issues that are of most importance to patients.

A small survey using the Attitudes to Randomised Trials Questionnaire (ARTQ) (Fallowfield *et al*, 1998) conducted in 1996 suggested that most patients were willing to consider trial participation. We administered the ARTQ to >1000 cancer patients being treated by multidisciplinary teams (MDT) in Wales. These data are a component of our large prospective study funded by Cancer Research UK examining multidisciplinary team members’ communication and involvement in clinical trials. The main study examines different aspects of trial recruitment, including (1) involvement of individual team members in clinical trials, (2) clarity of the communication by health professionals about clinical trials assessed by patients recruited to trials and (3) the attitudes of both patients and clinicians towards RCTs. The recruitment of patients to individual teams’ trial portfolios were audited before the study intervention, that is, attendance at customised MDT workshops. The attitudes of patients to RCTs were collected for each team to provide an evidence-based argument for them to consider approaching more patients about trials. This paper reports those data.

## Materials and methods

### Questionnaire

The seven-item ARTQ (Fallowfield *et al*, 1998) measures a positive or negative inclination towards (1) medical research in general; (2) a personal willingness to be involved in research; and (3) personal involvement in research involving randomisation. It is a self-report questionnaire, available in English or Welsh. The first three questions (see also Appendix 1) distinguish those who would consider joining a clinical research study from those who are uncertain or disinclined. Patients were asked to respond ‘Yes’, ‘No’ or ‘Do Not Know’ to these first three questions. Those who were uncertain or disinclined about trial entry then read three statements that described why the doctors want to do the study, the patients’ right to withdraw at any time and the treatments and side effects associated with the trial drugs. Participants responded to each of the statements consecutively and then answered a seventh and final question that asked if knowing the extra information contained in the statements would now encourage them to reconsider participation. Demographic details such as age, sex, cancer site and whether or not the patient had previous trial experience were also collected.

### Sample

For each of the 22 MDTs participating in the larger communication study, we gathered data from ∼50 patients per team attending surgical and oncology clinics from May 2007 to November 2009. The clinic staff helped identify suitable patients (namely, those not about to receive bad news about a diagnosis or recurrence – we do not have a record of how many participants fell into this category) and researchers gave them information sheets about the study.

Consenting patients completed the ARTQ while they waited for their clinical consultation, or at home and returned it in a prepaid envelope. The study had full ethical approval (South East Wales Local Research Ethics Committee Ref: 07/WSE03/17).

### Statistical analysis

Analyses of the relationship between dichotomised (‘Yes’ *vs* ‘Do Not Know’ and ‘No’) responses to individual questions and individual or trial-specific characteristics (such as age and cancer site) were conducted using logistic regression. The pattern of answers for all patients was displayed in a parallel coordinates plot. Additionally, patients were categorised into one of four ordered categories, according to how they answered the crucial questions 3 and 7. Category 1 contained all individuals who answered ‘Yes’ to question 3; categories 2–4 contained, respectively, the ‘Yes’, ‘Do Not Know’ and ‘No’ answers to question 7. This four-category response was modelled using the continuation ratio approach to ordinal data. Initial data exploration and all subsequent analyses were carried out using the R statistical package (R Foundation for Statistical Computing, Vienna, Austria).

## Results

Of the 1146 patients approached about the study, 1070 (93%) chose to take part. Four of these did not complete the second page of questions, leaving 1066 questionnaires suitable for analysis. The sample contained more women (654) than men (412), but the age distribution was comparable between sexes. More than half of the women had breast cancer (336 out of 654). Table 1 shows the characteristics in terms of age, sex, cancer type and previous trial participation.

Table 2 shows the responses given by the 1066 patients to each of the seven questions in the ARTQ. The first question (‘Do you think patients should be asked to take part in medical research?’) was widely supported, with 967 (91%) of patients answering ‘Yes’. Approximately 70% of patients (781) stated that they would be willing *personally* to participate in medical research (replying ‘Yes’ to ‘Would you be prepared to take part in a study comparing different treatments?’), with the remainder largely undecided. However, the ‘Yes’ figure fell to just over half (589) when asked ‘Would you be prepared to take part in a study where treatment was chosen at random?’ (question 3). The 477 patients who answered ‘No’ or ‘Do Not Know’ to question 3 completed questions 4–7. As further information was given, there was a gradual increase in the proportion of favourable attitudes towards trial participation (Table 2).

**Figure 1:**
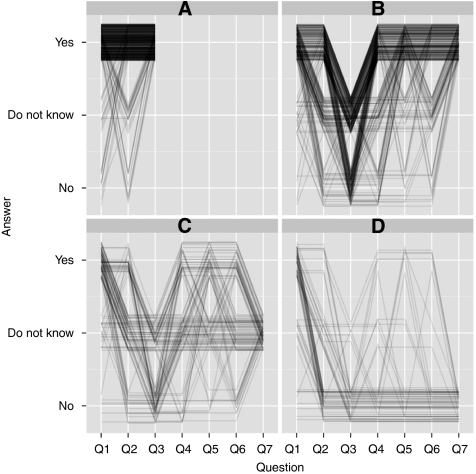
Parallel coordinates plot. (**A**) Of the patients, those (589, 55%) who were prepared to take part in a study where treatment was chosen at random are shown. (**B**) Those 297 (28%) patients who, despite not being prepared to take part in a study where treatment was chosen at random (said ‘No’ to Q3), subsequently would agree to participate if they knew that the following were taken into account: (1) that either treatment was completely suitable, (2) that they could leave the study if the treatment did not suit them, and (3) that there is plenty of information before the random choice was made. (**C**) Of the patients, 114 (11%) who remained uncertain about trial entry even with those factors in place. (**D**) Of the patients, 66 (6%) patients for whom the concept of a randomised trial is unacceptable and would refuse trial entry despite all further information.

Concerns about randomisation are a notable feature of Figure 1, a parallel coordinate plot of individuals’ responses to the seven questions, divided for visual convenience into the four groups previously mentioned (based on replies to questions 3 and 7). For instance, in the top left panel, lines that begin at ‘Yes’, drop to ‘No’ before returning to ‘Yes’ represent patients who answered ‘Yes’, ‘No’ and ‘Yes’ to questions 1–3, respectively. Because such patients were not required to answer questions 4–7, their lines end at this point. The other three panels display the responses of all those who did not answer ‘Yes’ to question 3, and hence continue into answers to questions 4–7. Among the 297 patients who ultimately answered ‘Yes’ to question 7 (top right panel), it is clear from Figure 1 that the majority (172, 58%) had difficulties only with the randomisation aspect of participation in medical research (question 3).

We explored whether some of the variation in individual response trajectories seen in Figure 1 could be explained by demographic or disease-specific information. In univariate logistic regression, men were significantly more likely to say ‘Yes’ to both questions 2 and 3 than women were. The estimated odds ratios were 1.52 and 1.51, respectively, with both *P*-values <0.01. However, men were not significantly more likely to be encouraged to consider trial entry by the statements and questions on the second page of the questionnaire. In contrast, age was a strong predictor of being more encouraged by the statements surrounding questions 4–7; both older (⩾80) and younger (⩽35) patients undecided or disinclined at question 3 were less likely to agree to participate (global *P*-values all <0.03). At question 3 itself, those >70 years of age were significantly less likely than their younger counterparts to give a ‘Yes’ answer (*P*-value <0.01).

Unsurprisingly, those who had previous trial experience were significantly more likely to agree to personal involvement and be positively inclined towards future randomised trials (*P*-values <0.01 for questions 1–3). In fact, of the 210 individuals with trial experience, 162 (77%) said they would be willing to agree to participate in a randomised trial again (question 3). Among the 48 who were disinclined or unsure, there was no consistent indication that previous participation made them any more or less likely to accept participation upon reaching question 7.

Like age, cancer site was significantly related to initial inclination towards trials (*P*-values of 0.04 and <0.01 for questions 2 and 3), but not obviously connected to the likelihood of being convinced (questions 4–7). The biggest individual effect was seen in the gynaecological group, who were less likely to give a ‘Yes’ answer to question 3 (their estimated odds were 61% smaller than the reference category of haematological cancers and lymphoma).

### Multivariate analysis

The division of patients according to their answers to questions 3 and 7 allows us to conduct a summary analysis of the variability in patient attitudes. Results from modelling the ordinal outcome as a function of age, sex, cancer site, previous trial participation and study region are shown in the first set of columns of Table 3 . As no evidence that study region was an important indicator of attitude was found, the model was refitted with these terms removed, as shown in the second set of columns of Table 3. All other variables retain strong statistical significance in global tests.

In particular, the odds of being in a group more favourably inclined towards trials are nearly tripled if a patient has previously participated in a trial, and are increased by 67% if the patient is male. Lung and gynaecological cancer patients had 23 and 26% smaller odds of being more favourably inclined than those with haematological cancer, whereas breast cancer patients had 23% higher odds.

To make this more concrete, consider four hypothetical patients. All are aged between 50 and 65 years, and all have either haematological malignancies or lymphoma. One is a woman not having previously participated in a trial; based on our ordinal model, her estimated probability of a ‘Yes’ answer (either at question 3 or at question 7) is 78%. The other woman has participated in an RCT before; her ‘Yes’ probability is 94%. The other two patients are men: one is trial naive, and his chance of a positive response is 88%. The final man has trial experience; his probability is 97%.

Now consider two other patients, both female and both aged between 50 and 65 years, neither of whom has previous trial experience. If one is a breast cancer patient, her chance of agreeing to participate is estimated by our model to be 82%. Should the other have a gynaecological cancer, her model-based probability of saying ‘Yes’ is just 70%.

## Discussion

This large survey revealed that the majority of patients with cancer (83%) are willing to consider participation in clinical trials. Just over half the patients approached were happy to enter even with the difficult concept of randomisation explained only minimally. Of the 45% (477 out of 1066) of patients who expressed unease at the prospect of randomisation, two-thirds (62% 297 out of 477) changed their minds and would consider joining when provided with further types of information that form part of the standard informed consent discussion described in good clinical practice guidelines. Specifically, they were told the logic for the trial and all treatments were suitable, they had the right to withdraw from the study and that they would be provided with information about the treatments and possible side effects. The results accord closely with the 1996 study, which involved a smaller cohort of patients with breast and urological cancer attending University College London and Royal Marsden hospitals (Fallowfield *et al*, 1998).

The finding that men were less likely than women to be dissuaded by the introduction of randomisation was unexpected. This result might reflect the willingness of men to take part in seemingly riskier activities; explanation of the scientific rationale for randomisation was not provided unless patients were undecided or said ‘No’. Deciding to participate in a randomised trial has an element of risk in that the participant would not know which treatment s/he would receive until after consenting to the RCT. Data show that women as a group of any age are more risk averse than men (Gustafson, 1998) and women find risky situations more stressful (Lighthall *et al*, 2009). More recently, Han *et al* (2009), in a survey involving >4000 members of the US public, reported that aversion to the uncertainty of risk information was associated with older age, lower socioeconomic education and female sex. One limitation of our study is that we did not gather any education or employment data; results such as the gender differences may possibly be explained in terms of other demographic variables.

Patients’ preference for and understanding of the term randomisation has been widely studied (Featherstone and Donovan, 1998, 2002). In one study, patients expressed a preference for descriptions of randomisation that gave more of the rationale, and a dislike for analogies, especially ‘coin-tossing’ (Jenkins *et al*, 2002). The use of the coin-tossing analogy to describe randomisation in the current study may have further discouraged patients to consider participation in the hypothetical two-treatment arm trial.

The fact that given additional information, so many of those who initially dissented or who were uncertain would be likely to consider RCTs demonstrates the importance of ensuring that these issues are not omitted from trial discussions. It is too easy to assume that the patient information sheets cover these areas satisfactorily. Apart from the fact that few patients read these and that reading them is highly correlated with education (Hietanen *et al*, 2000), many information sheets are written at too high a reading level (Paasche-Orlow *et al*, 2003) and are convoluted in style (Knapp *et al*, 2009).

In contrast, it is important to note that 6% (66 out of 1066) of patients were not prepared to consider entry into a randomised trial despite additional information, and a further 11% (114 out of 1066) remained uncertain. Data collected by the South Wales WCRN (Wales Cancer Research Network) regional centres for the period 2009–2010 revealed that 39% of patients (627 out of 1589) declined to participate in an RCT (WCRN, personal communication). These figures might be interpreted as showing that a greater number of patients in routine clinical practice than in hypothetical surveys will refuse RCTs, but establishing reasons for accepting or declining trial entry is important. A limitation of the results from our study is that patients were asked to consider a hypothetical scenario about trial entry, but not only does the ARTQ differentiate those patients who do and do not feel comfortable with the concept of randomisation, but also in a previous study it reliably predicted with 80% accuracy those patients who would go on to participate in a clinical trial (Fleissig *et al*, 2001).

Our results show that >80% of cancer patients might well be prepared to participate in randomised controlled trials. However, just under half of those will be initially put off by the concept of randomisation and will require further explanation to enable them to accept the concept of a trial and feel comfortable about consent. Among those who refuse trial entry in routine oncology practice, there is a group for whom such research is unacceptable. Different types of trial design, such as placebo control and multi arm, anxieties about side effects or the extra effort that some trials require may all be disincentives.

Although we should be aware of possible coercion in some trials in which very high (>90%) consent levels have been achieved, such figures could of course be because of attractive features of the trial. These include drugs or devices without the likelihood of severe adverse events, trials that have little extra effort or burdens, or the trial may be the only way in which patients can access a novel drug or procedure.

Our results contribute to an evidence base that patients’ attitudes are not the cause of low recruitment to trials. A significant minority (∼20%) have deeply held concerns and should not be coerced into unwilling participation in cancer or other randomised trials. However, the message for investigators and research staff is that most patients are willing to consider trial participation, although many require in-depth discussion to resolve fears raised by the concept of randomisation.

## Figures and Tables

**Table 1:** Patient characteristics, *n* (%)

	**Male (*n*=412)**	**Female (*n*=654)**
*Age group*		
20–35	7 (2%)	16 (2%)
36–50	29 (7%)	141 (22%)
51–65	172 (42%)	271 (42%)
65–80	183 (44%)	195 (30%)
80–85	20 (5%)	30 (4%)
Missing	1	1
		
*Cancer site*		
Breast	0 (0%)	336 (51%)
Upper and lower gastrointestinal (GI)	159 (39%)	92 (14%)
Lung	49 (12%)	24 (4%)
Gynaecological	0 (0%)	113 (17%)
Urinary	96 (23%)	2 (0%)
Haematological	108 (26%)	87 (13%)
		
*Previous trial experience*		
Yes	75 (18%)	135 (21%)
No	317 (77%)	496 (76%)
Unsure	20 (5%)	23 (3%)

**Table 2:** Responses to questions 1–7

**Question**	**Yes**	**No**	**Do not know**
Q1. Do you think that patients should be asked to take part in medical research?	967 (91%)	29 (3%)	70 (6%)
*Suppose that you were asked to take part in a research study comparing two treatments, both of which were suitable for your illness*.			
Q2. Would you be prepared to take part in a study comparing different treatments?	781 (73%)	98 (9%)	187 (18%)
*Usually, the only scientific way to compare one treatment with another is for the choice between the two to be made randomly, rather like tossing a coin.*			
Q3. Would you be prepared to take part in a study where treatment was chosen at random?	589 (55%)	208 (20%)	269 (25%)
*In a randomised study, a choice would be made between two treatments, either of which would be suitable for you. Your doctor and experts in the field do not know for sure if one treatment is better than the other or if they are both the same, that's why they want to do the study.*			
Q4. Would knowing that encourage you to take part?	244 (51%)	102 (21%)	131 (28%)
			
*In a random choice study, if the treatment you were receiving did not suit you for any reason, you could always leave the study. Your doctor would then give you whatever other treatment might be appropriate for you.*			
			
Q5. Would that encourage you to take part?	305 (64%)	78 (16%)	94 (20%)
			
*Before you agreed to enter a random choice study, the doctor would tell you all about the two treatments being compared, including any side effects before you were allocated to one or the other.*			
			
Q6. Would that encourage you to take part?	282 (59%)	83 (17%)	112 (24%)
			
*If you knew that the following were taken into account: (a) that either treatment was completely suitable; (b) that you could leave the study if the treatment did not suit you; and (c) that there is plenty of information before the random choice was made*			
			
Q7. Would all these things together mean that you would change your mind and agree to take part?	297 (62%)	66 (14%)	114 (24%)

**Table 3:** Ordinal regression table

	**OR**	**95% CI**	***P*-value**	**OR**	**95% CI**	***P*-value**
(Intercept)	1.30	(0.61, 2.89)	0.51	1.24	(0.60, 2.68)	0.58
Cohortordinal⩾2	1.58	(1.25, 1.98)	<0.01	1.57	(1.25, 1.98)	<0.01
Cohortordinal⩾3	1.82	(1.30, 2.56)	<0.01	1.81	(1.30, 2.56)	<0.01
Cohort (global)			<0.01			<0.01
Agegp(35,50)	0.92	(0.42, 1.95)	0.83	0.93	(0.42, 1.97)	0.85
Agegp(50,65)	0.71	(0.33, 1.47)	0.36	0.72	(0.33, 1.48)	0.38
Agegp(65,80)	0.63	(0.29, 1.30)	0.22	0.64	(0.29, 1.32)	0.23
Agegp(80,95)	0.44	(0.19, 1.00)	0.05	0.45	(0.19, 1.01)	0.06
Agegp (global)			<0.01			<0.01
Sexm	1.67	(1.25, 2.23)	<0.01	1.67	(1.25, 2.23)	<0.01
Cancersitebreast	1.18	(0.83, 1.66)	0.35	1.23	(0.89, 1.72)	0.21
Cancersitecolo+uppergi	0.97	(0.69, 1.35)	0.84	0.95	(0.69, 1.32)	0.77
Cancersitegynae	0.75	(0.50, 1.12)	0.16	0.74	(0.50, 1.09)	0.13
Cancersitelung	0.75	(0.48, 1.17)	0.20	0.77	(0.50, 1.19)	0.24
Cancersiteurology	0.83	(0.53, 1.29)	0.40	0.83	(0.54, 1.29)	0.41
Cancersite (global)			0.10			0.04
Prevpartnot sure	0.62	(0.39, 0.99)	0.05	0.61	(0.39, 0.97)	0.04
Prevpartyes	2.81	(2.07, 3.87)	<0.01	2.78	(2.05, 3.80)	<0.01
Prevpart (global)			<0.01			<0.01
Regionse	0.91	(0.71, 1.17)	0.45			
Regionsw	1.04	(0.76, 1.41)	0.82			
Region (global)			0.58			

Abbreviations: CI=confidence interval; OR=odds ratio.
